# Why only one-quarter of placental fetal growth restriction triggers preeclampsia? Clinical risk factors in 2060 cases in a 24-year cohort of 90,000 births

**DOI:** 10.1371/journal.pone.0354687

**Published:** 2026-09-18

**Authors:** Pierre-Yves Robillard, Gustaaf Dekker, Nandor Gabor Than, Francesco Bonsante, Malik Boukerrou, Marco Scioscia, Phuong Lien Tran, Silvia Iacobelli

**Affiliations:** 1 Service de Néonatologie, Centre Hospitalier Universitaire Sud Réunion, Saint-Pierre Cedex, La Réunion; 2 Centre d’Etudes Périnatales Océan Indien (CEPOI). Centre Hospitalier Universitaire Sud Réunion, Saint-Pierre cedex, La réunion; 3 Department of Obstetrics & Gynaecology, University of Adelaide, Robinson Institute, Lyell McEwin Hospital, Adelaide, Australia; 4 Systems Biology of Reproduction Research Group, Institute of Molecular Life Sciences, HUN-REN Research Center for Natural Sciences, Budapest, Hungary; 5 Department of Obstetrics and Gynaecology, School of Medicine, Semmelweis University, Budapest, Hungary; 6 Service ‌‌de Gynécologie et Obstétrique. Centre Hospitalier Universitaire Sud Réunion, Saint-Pierre cedex, La réunion; 7 Department of Obstetrics and Gynaecology, Mater Dei Hospital, Bari, Italy; University of Lausanne: Universite de Lausanne, SWITZERLAND

## Abstract

**Objectives:**

To compare risk factors between preeclampsia-associated fetal growth restriction (PFGR) and non-preeclamptic vascular FGR (VFGR) in a cohort of 96,094 consecutive singleton non-malformed pregnancies (≥22 weeks gestation) to identify susceptibility and protective factors differentiating maternal tolerance from overt hypertensive disease.

**Design:**

Observational population-based cohort study over 24.5 years at a university maternity center.

**Results:**

Among 94,435 non-malformed births, 9,356 (10.0%) were SGA, with 2,060 (22%) classified as FGR based on abnormal Doppler indices: 529 PFGR (25.7%) and 1,531 VFGR (74.3%). VFGR predominated in late-onset cases (≥34 weeks; 78% vs. 46% early-onset <34 weeks). Compared to VFGR, PFGR mothers were older (mean 28 vs. 27 years), had higher pre-pregnancy BMI (25.9–26.8 vs. 24.0–24.4 kg/m^2^), and greater gestational weight gain (by 1–3 kg). Obesity (BMI ≥ 30 kg/m^2^) increased PFGR risk (OR 1.4–2.1), while underweight (BMI < 18.5 kg/m^2^) (OR 0.36–0.50) and smoking (OR 0.36–0.51) was protective for PFGR. Chronic hypertension (OR 2.8–3.0), previous preeclampsia (OR 3.3–9.9 in multiparas), primipaternity (OR 2.1–6.8), and renal disease (OR 5.0–7.8) were key risks for PFGR, with stronger effects in early-onset. Neonatal outcomes were similarly poor in early-onset PFGR/VFGR but worse in late-onset PFGR (e.g., higher prematurity, fetal death). Multivariable models confirmed independent associations of PFGR with age, BMI, chronic hypertension, primipaternity, previous abortion, and smoking (protective).

**Conclusions:**

This study reveals VFGR as the majority of placental-mediated FGR, with PFGR distinguished by metabolic, vascular, and immunological risks. These insights prioritize research into maternal protective mechanisms, potentially enabling targeted predictions and interventions to mitigate FGR’s impacts on mothers and offspring.

## Introduction

By international consensus, the 10th percentile birthweight was adopted in the 1960s as the threshold for defining small-for-gestational-age newborns (SGA) [1], later reaffirmed by the World Health Organization in 1995 [2]. However, SGA is not synonymous with fetal growth restriction (FGR), which reflects a pathological slowing or arrest of fetal growth velocity during pregnancy [3]. Constitutionally small but healthy SGA infants must therefore be distinguished from FGR-SGA newborns, who, unlike normal SGA infants, are at increased risk of morbidity, mortality, intrauterine malnutrition, and a wasted phenotype [3,4]. This distinction requires consideration of maternal characteristics such as height, weight, parity, and ethnicity [3,4]. In placenta-mediated FGR, fetal growth is initially normal before progressively decelerating in utero, with diagnosis generally based on serial ultrasound examinations. Since the pioneering studies of the 1980s and 1990s [5–9], later summarized by Bamberg and Kalache [8], Doppler velocimetry, including uterine artery assessment, increased umbilical artery pulsatility index, and reduced middle cerebral artery pulsatility index; has become the reference standard for differentiating constitutional SGA from true FGR. It has long been recognized that fetal growth restriction may occur in the absence of gestational hypertension or clinically overt preeclampsia, reflecting placental dysfunction without maternal hypertensive manifestations [10–14].

**Fig 1:**
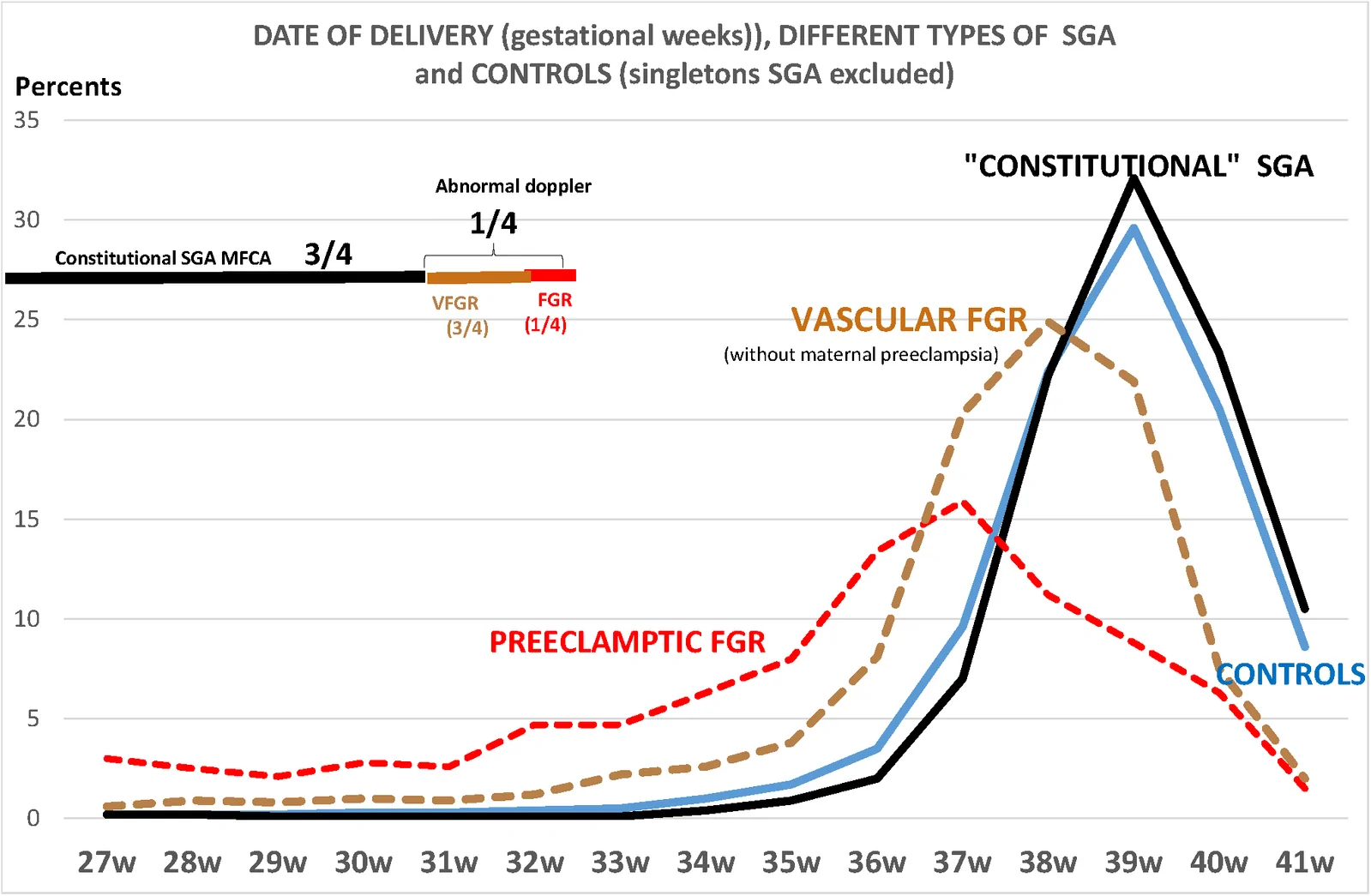
Distribution of the different categories of small-for-gestational-age (SGA) newborns in the cohort of 94,435 non-malformed singleton pregnancies. The curves represent gestational age at delivery for preeclampsia-associated fetal growth restriction (PFGR, N = 529), vascular fetal growth restriction without maternal preeclampsia (VFGR, N = 1,531), constitutional SGA (ConstS, N = 7,296), and controls without SGA (N = 80,829). The horizontal line displayed at the top of the figure is a schematic summary of the observed proportions within the SGA population: approximately three-quarters of SGA newborns were classified as constitutional SGA, whereas one-quarter presented Doppler abnormalities consistent with fetal growth restriction (FGR). Among these FGR cases, approximately three-quarters corresponded to VFGR and one-quarter to PFGR.

Concerning constitutionally small Damhuis et al evaluate their prevalence at approximately 40% of babies with a fetal size less than the 10th percentile are [15], while Osuchukwu & Reed, evaluate this rate around 50% [4]. Unexpectedly, in a recent study [16], we described in a large population of 83,917 non-malformed singleton pregnancies that 8,601 infants (10.2%) were small for gestational age (SGA), from which two major findings emerged, see Fig 1. First, the majority (77.6%; n = 6,676) of SGA infants were constitutionally small, with no evidence of placental vascular or Doppler abnormalities during pregnancy, and specific morbidity/mortality (except intra-uterine fœtal deaths as compared to non-SGA pregnancies serving as controls). Second, only 22.4% of SGA cases represented true fetal growth restriction (FGR) due to placental vascular insufficiency (abnormal Doppler findings from the second trimester onward and impaired materno-fetal exchange, mainly in the third trimester). Surprisingly, among these placental-mediated FGR cases, only about one-quarter occurred in mothers who developed preeclampsia. The remaining three-quarters of FGR pregnancies were carried by mothers who never manifested clinical symptoms of preeclampsia. This observation directly challenges the classic textbook statement that “the most common cause of FGR in a structurally normal fetus is maternal vascular malperfusion associated with preeclampsia” [8]. Had all placental-mediated FGR cases triggered maternal preeclampsia, the incidence of preeclampsia would have reached 10–15% (and eclampsia 3–4%), which is clearly not observed in reality. From an evolutionary medicine perspective, the fact that most severe placental insufficiency does not provoke life-threatening maternal hypertensive disease suggests that human pregnancy has evolved powerful protective mechanisms to shield the mother from preeclamptic/eclamptic complications, even at the cost of fetal harm.

Consequently, in 2026 and beyond, a research priority should be to understand why, in the presence of virtually identical placental lesions, some women develop preeclampsia (PFGR) while the majority tolerate the same or similar degree of vascular FGR (VFGR) without maternal syndrome. The aim of the present study is therefore to compare clinical risk factors between preeclampsia-associated FGR (PFGR) and “pure” vascular FGR without preeclampsia (VFGR) to identify protective or susceptibility factors which may guide future genetic, epigenetic, and multi-omics research toward the most promising pathways.

## Materials and methods

From January 1^st^, 2001, to June 30, 2025 (24.5 years), the hospital records of all women who gave birth at the maternity of the University of South Reunion were abstracted in a standardized fashion. The study sample was drawn from the hospital perinatal database which prospectively records data of all mother-infant pairs since 2001. Information is collected at the time of delivery and at the infant hospital discharge and regularly audited by appropriately trained staff. This epidemiological perinatal data base contains information on obstetrical risk factors, description of delivery, and maternal and neonatal outcomes. For the purpose of this study, records have been validated and have been used anonymously. All pregnant women in Reunion Island as part of the French National Health Care System have their prenatal visits, biological and ultrasound examinations, at the time of the morphology scan and anthropological characteristics recorded in a maternity booklet and access to maternity care free of charge as provided by the French healthcare system, which combines freedom of medical practice with nationwide social security. Hospitals have European standards of care health care, in particular maternity services are based on scheduled appointments (8 prenatal visits and on average 4 ultrasounds).

### Design and study population

The maternity department of Saint Pierre hospital is a tertiary care centre that performs about 4,300 deliveries per year, thus representing about 80% of deliveries of the Southern area of Reunion Island, and it is the only level-3 maternity (the other maternity is a private clinic, level 1). Reunion Island is a French overseas region in the Southern Indian Ocean.

**Definition of exposure and outcomes**. During the 23.5-year period all consecutive singleton pregnancies after 22 weeks gestation have been analysed.

PREECLAMPSIA, gestational hypertension and eclampsia were diagnosed according to the definition issued by the International Society for the Study of Hypertension in Pregnancy (ISSHP) relatively to the guidelines in force at the year of pregnancy [4]. Early onset preeclampsia is defined as preeclampsia resulting in birth before 34 weeks of gestation, late onset preeclampsia at 34 weeks and onward.

PFGR: SGA with diagnosed abnormal Doppler conditions on at least 2 different examinations 2 weeks apart associated with maternal diagnosis of preeclampsia or HELLP syndrome:

VFGR All foetuses with diagnosed abnormal Doppler conditions and labelled “FGR” (Fetal growth restriction) confirmed abnormal growth trajectory, and with NO maternal preeclamptic disease. ALL coding have been made by either a midwife or a physician. We searched in all the following items the unique indication “FGR”.

- Indication of labor induction (26 possible coding)
- Indication of caesarean section (35 possible coding)
- Indication of amniocentesis (15 possible coding)
- Indication of hospitalization (at the at risk clinic, 43 possible coding, or day care hospitalization and prenatal diagnosis, 45 possible coding)

ConstS: constitutional SGA at birth without all the preceding conditions (PES, VascS)

CONTROLS have been defined by all normotensive singleton pregnancies comprising newborns from the 10^th^ to the 100^th^ centile (SGA excluded, malformations excluded).

Chronic hypertension was defined as known hypertension before pregnancy and/or hypertension ≥140 mmhg (systolic)or ≥ 90 mmhg (diastolic) before the 20th week of gestation. These cases correspond to superimposed preeclampsia

Levels of education: none, primary school, « collège » in the French system (6th to 9th grade), « lycée » in the French system (10th to 12th grade), University: education after high scool.

Smoking is a qualitative item « yes/no » in the perinatal database (direct inquiry with the mothers).

Primipaternity in multiparas: new paternity for the index pregnancy (direct inquiry with the mother).

### Statistical analysis

Data are presented as numbers and proportions (%) for categorical variables and as mean and standard deviation (SD) for continuous ones, as appropriate. Comparisons between groups were performed using χ^2^-test and odds ratio (OR) with 95% confidence interval (CI) was also calculated. Paired t-test was used for parametric and the Mann-Whitney *U* test for non-parametric continuous variables. P-values <0.05 were considered statistically significant. Epidemiological data have been recorded and analysed with the software EPI-INFO 7.1.5 (2008, CDC Atlanta, OMS), EPIDATA 3.0 and EPIDATA Analysis V2.2.2.183. Denmark

To validate the independent association of maternal age and other confounding factors on different sorts of SGA we built a multiple regression logistic model. Variables associated with all kinds of SGA in bivariate analysis, with a p-value below 0.1 or known to be associated with the outcome in the literature were included in the model. A stepwise backward strategy was then applied to obtain the final model. The goodness of fit was assessed using the Hosmer-Lemeshow test. A p-value below 0.05 was considered significant. All analyses were performed using MedCalc software (version 12.3.0; MedCalc Software’s, Ostend, Belgium).

We considered the following covariates as possible confounders in this analysis with the outcome PES, VascS, constitutional -SGA: maternal age by increment of 5 years of age, ore-pregnancy BMI by increment of 5 kg/m², smoking during pregnancy, chronic hypertension, primiparity, gestational weight gain. Among multiparas: previous preeclampsia, previous SGA and “primipaternity”. We included these variables and calculated the χ² for trend (Mantel extension), the odds ratios for each exposure level compared with the first exposure level.

## Results

On the entire 24.5-year singleton-pregnancies’ cohort of N = 96,084, we excluded all kinds of foetal or neonatal malformations (N = 2649, 2.8% of the singleton cohort) including foetopathies due to CMV, rubella, parvo-virus infections, foetal alcoholic syndrome (18% of SGA in this small population of malformed fetuses). The remaining non-malformed SGA population was 9,356/94,435 (10.0%). Among these 9,356 non-malformed SGA newborns, 7,296 were constitutional SGA (without Doppler anomalies during pregnancy). Those with documented Doppler anomalies therefore coined foetal growth retardation (FGR). FGR associated with maternal preeclampsia (PFGR, N = 529) represented 5.7% of all SGA cases, those without maternal preeclampsia but diagnosed with having abnormal Doppler indices during the pregnancy follow-up-, « vascular FGR » (VFGR, N = 1,531), represented 16.3% of all SGA.

The final study population is composed of 2,060 FGR; 529 PFGR (25.7% of all FGR) and 1,531 VFGR (74.3% of all FGR). First, it is noteworthy that while VFGR represents globally ~75% of FGR cases, early-onset VFGR (< 34 weeks gestation) represents only 46% of early-onset cases (103/223) while they represent 78% of late cases (34^th^ week gestation onward, 1428/1827).

As shown in Table 1, there were no differences between early-onset and late-onset cases of preeclamptic (PFGR) and vascular FGR (VFGR) for average gestity & parity, primiparity, grand parity (5 children and plus), rate of Being younger than 18 years was indifferent in early-onset cases, but a tendency to be protective for preeclampsia was seen in late-onset cases (OR = 0.54, p = 0.07). While being 35 years of age or plus had ~ 40% increased risk for preeclamptic cases (OR 1.4, o = 0.009 in late cases, OR 1.8, p = 0.12 in early ones). Ten years of education was indifferent in early-onset cases, but was protective for preeclampsia in late-onset cases (OR 0.72, p = 0.005).

**Table 1:** Maternal characteristics. FGR.// 34^th^ week gestation EARLY ONSET& EOP N = 223// LATE ONSET & LOP N = 1837.

	PFGR preeclamptic N = 120 (%)	VFGR (Non-PE) N = 103 (%)	OR [95% CI]	P value	PFGR preeclamptic N = 409 (%)	VFGR (Non-PE) N = 1428 (%)	OR [95% CI]	P value
**Maternal age (years: mean ± sd)**	28.4 ± 6.9	27.2 ± 6.6		0.19	28.3 ± 7.2	27.4 ± 6.6		0.01
**Gestity ± sd**	2.7 ± 1.83	2.7 ± 2.2		0.91	2.5 ± 1.7	2.6 ± 1.8		0.48
**Parity ± sd**	1.1 ± 1.6	1.03 ± 1.4		0.69	0.97 ± 1.3	0.98 ± 1.4		0.98
**primiparous women**	62 (48.3)	58 (51.7)	1.05 [0.6-1.8]	0.86	209 (51.1)	691 (48.4)	1.11 [0.89-1.4]	0.33
**Adolescents < 18 years**	6 (5.0)	4 (3.9)	1.3 [0.36-4.8]	0.69	10 (2.4)	63 (4.4)	0.54 [0.28-1.07]	0.07
**Age ≥ 35 years**	23 (19.2)	12 (11.7)	1.8 [0.85-3.8]	0.12	93 (22.7)	244 (17.1)	1.4 [1.09-1.9}	0.009
**Grand multips (≥ 5)**	9 (7.5)	7 (6.8)	0.98 [0.4-3.1]	0.96	24 (5.9)	79 (5.5)	1.06 [0.66-1.7]	0.79
**Education 10th grade** **or over**	72 (65.5)	59 (61.5)	1.19 [0.67-2.1]	0.55	222 (56.8)	886 (64.6)	0.72 [0.57-0.9]	0.005
**Living single**	50 (41.7)	46 (44.7)	0.89 [0.52-1.5]	0.65	174 (42.8)	605 (42.6)	1.01 |0.8-1.3]	0.95
**BMI (mean ± sd)**	26.8 ± 6.0	24.0 ± 5.8		0.009	25.9 ± 6.2	24.4 ± 6.3		0.0001
**Obesity ≥ 30 kg/m²**	29/94 (30.9)	17/97 (18.1)	2.1 [1.06-4.2]	0.03	94/388 (24.2)	261/1394 (18.7)	1.4 [1.06-1.8]	0.02
**Thin < 18.5 kg/m²**	17/94 (18.1)	24/97 (28.2)	0.36 [0.33-1.4]	0.26	62/388 (16.0)	382/1394 (27.4)	0.50 [0.37-0.68	<0.0001
**Smoking**	15 (12.5)	29 (28.2)	0.36 [0.18-0.73]	0.003	52 (12.7)	317 (22.2)	0.51 [0.37-0.70]	<0.0001
**MULTIPARAS**	**N = 76**	**N = 65**			**N = 257**	**N = 923**	**//////////////**	**////////////**
**NEW PATERNITY**	15 (19.7)	13 (20.0)	0.98 [0.4-2.3]	0.96	48 (18.7)	186 (20.2)	0.91 [0.64-1.3]	0.60
**History of elective abortion**	23 (30.3)	16 (24.6)	1.33 [0.63-2.8]	0.43	56 (21.8)	292 (31.6)	0.60 [0.43-0.83]	0.002
**History of miscarriage**	26 (34.2)	27 (41.5)	0.73 [0.37-1.5]	0.37	85 (33.1)	270 (29.3)	1.2 [0.89-1.6]	0.24

Preeclamptic PFGR women were older than vascular ones by an average of one year (28 vs 27 years) in both early- and late-onset cases. Preeclamptic women were in average heavier by 2.8 kg/m^2^ in early-onset cases (26.8 vs 24.0, p = 0.009), and heavier by 1.5 kg/m^2^ in late-onset cases (25.9 vs 24.4, p = 0.0001).

Obesity (≥30 kg/m^2^ was a strong risk factor for preeclampsia both in early-onset (OR 2.1, p = 0.03) and late-onset cases (OR = 1.4, p = 0.02). Conversely, being underweight (< 18.5 kg/m^2^) was protective for preeclamsia (OR 0.50, p= < 0.0001) in late-onset cases with also a tendency for early-onset ones (OR 0.36, p = 0.12).

Smoking was a strong protective factor for preeclamptic PFGR as well both in early-onset and late-onset (respectively OR 0.36, p = 0.003, and OR 0.51, p= < 0.0001).

History of volunteer abortion was indifferent for early-onset cases, but was protective for late-onset ones (OR 0.60, p = 0.002, while history of miscarriages were similar in all cases.

As can be seen in Table 2, there were no differences between early-onset and late-onset cases of preeclamptic (PFGR) and vascular cases (VFGR) for gestational diabetes, history of perinatal death, and corticoid treatment for foetal lung maturation. Hospitalizations at the at-risk clinic were similar in preeclamptic and vascular FGR cases (over 80%) in the early-onset group, while it was much higher in preeclamptic cases at the end of pregnancies (OR 3.1, p < 0.0001.

**Table 2:** Pregnancy characteristics.// 34^th^ week gestation. EARLY ONSET& EOP N = 223// LATE ONSET & LOP N = 1837.

	PFGR preeclamptic N = 120 (%)	VFGR (Non-PE) N = 103 (%)	OR [95% CI]	P value	PFGR preeclamptic N = 409 (%)	VFGR (Non-PE) N = 1428 (%)	OR [95% CI]	P value
**Gestational diabetes**	10 (8.5)	7 (6.9)	1.2 [0.46-3.4]	0.56	64 (15.7)	195 (13.8)	1.17 [[086-1.6]	0.32
**Chronic hypertension**	19 (15.8)	6 (5.8)	3.0 [1.2-8.0]	0.018	22 (5.4)	28 (2.0)	2.8 [1.3-5.0]	0.0002
**Pregnancies & pathologies** **• Asthma** **• Thyroid** **• Renal pathologies**	14 (11.8) 3 (2.5) 2 (1.7)	4 (3.9) 0 (0) 2 (1.9)	3.3 [1.08-11] -- 0.86 [0.12-6]	0.03 -- 0.87	26 (6.3) 11 (2.7) 7 (1.7)	135 (9.5) 19 (1.3) 5 (0.4)	0.6 [0.4-0.99] 2.0 [1.0-4.3] 5.0 [1.6-15.7]	0.05 0.05 0.002
**Hospitalizations in at risk clinic**	99 (82.5)	87 (84.5)	0.87 [0.4-1.8]	0.69	231 (56.5)	420 (29.4)	3.1 [2.5-3.9]	< 0.0001
**Corticoid treatment** **(fœtal maturation)**	70 (58.3]	59 (57.3)	1.04 [0.6-1.8]	0.87	40 (9.8)	115 (8.1)	1.2 [0.85-1.8]	0.27
**Mother in intensive care**	7 (5.8)	0 (0)	--	--	10 (2.5)	1 (0.1)	35 [4.6-280]	< 0.0001
**MULTIPARAS**	**N = 76**	**N = 65**			**N = 257**	**N = 923**		
**History of preeclampsia**	18 (23.8)	2 (3.1)	9.9 [2.5-45]	0.0009	23 (8.9)	25 (2.7)	3.3 [1.9-5.4]	< 0.0001
**History of previous SGA**	2 (2.6)	1 (1.5)	1.7 [0.16-20.3]	0.65	9 (3.5)	68 (7.4)	0.45 [0.22-0.9]	0.02
**History perinatal deaths**	6 (8.0)	8 (12.7)	0.60 [1.4-3.0]	0.36	13 (5.1)	59 (6.5)	0.78 [0.42-1.4]	0.42

Chronic hypertension was 3 times higher in preeclamptic cases in early-onset (OR 3.0, p = 0.02) and late-onset FGR (OR 2.8, p = 0.0002). History of previous preeclampsia in multiparas was an enormous risk factor for preeclampsia both in early-onset (OR 9.9, p = 0.0009) and late-onset FGR (OR 3.3, p < 0.0001).

Preexisting maternal renal pathologies were strong risk factors for preeclampsia in late-onset cases (OR 5.0, p = 0.002) but not for early-onset cases (p = 0.87). Thyroid pathologies were a moderate risk factor for preeclampsia in both the early-onset and late-onset groups. Asthma was controversial: a strong risk factor for PFGR in early-onset cases (OR 3.3, p = 0.03, but protective for late-onset cases (OR 0.6, p = 0.05).

Interestingly, concerning the foetal/neonatal outcomes in early-onset cases, the results were identically very bad for PFGR and VFGR in quite all aspects (Table 3). This was true for the. rate of c-sections, post-partum hemorrhage, very low birthweights (<1500g), neonatal morbidities (HMD, bad Apgar scores…), in utero foetal deaths, except that preeclamptic FGR had higher rates of grand prematurity ≤32 weeks gestation (OR 2.45, p= < 0.0001 and delivery terms 30.7 weeks vs 31.3 weeks, p = 0.05. In late-onset cases, foetal/neonatal outcomes concerning foetal growth restricted SGAs, results were constantly more catastrophic for all the items in case of maternal preeclampsia.

**Table 3:** Some Delivery and Neonatal characteristics (liveborn infants).// 34^th^ week gestation. EARLY ONSET& EOP N = 200// LATE ONSET & LOP N = 1825.

	PFGR preeclamptic N = 97 (%)	VFGR (Non-PE) N = 83 (%)	OR [95% CI]	P value	PFGR preeclamptic N = 400 (%)	VFGR (Non-PE) N = 1425 (%)	OR [95% CI]	P value
**Rate C-section (%)**	97 (100)	81 (97.6)		0.12	198 (49.5)	398 (27.9)	2.5 [2.0-3.2]	< 0.0001
**Induced deliveries (%)**	1	0		0.35	252 (63.0)	921 (64.6)	0.93 [0.74-1.2]	0.54
**Post partum hemorhhage#**	2/82 (2.4)	1/67 (1.5)	1.7 [0.15-18]	0.68	27/343 (7.9)	21/1233 (1.7)	4.9 [2.8-8.8]	< 0.0001
**Fetal sex Girl**	46 (47.4)	35 (42.2)	1.2	0.48	219 (54.8)	772 (54.2)		0.83
**Birth weight (g)**	982 ± 280	1037 ± 262		0.17	2094 ± 387	2226 ± 321		< 0.0001
**Low birth weight < 2500g**	97 (100)	83 (100)		–	326 (81.5)	1125 (78.9)	1.17 [0.89-1.6]	0.26
**Very low birth weight <1500gr**	97 (100)	83 (100)		–	23 (5.8)	39 (2.7)	2.1 [1.3-3.7]	0.003
**Delivery term (weeks):**	30.7 ± 2.1	31.3 ± 1.9		0.05	37.0 ± 1.8	37.8 ± 1.4		< 0.0001
**Preterm birth <37 weeks**	97 (100)	83 (100)		–	158 (39.5)	216 (15.2)	3.6 [2.9-4.7]	< 0.0001
**Preterm birth <33 weeks**	78 (80.4)	52 (62.7)	2.45 [1.25-4.8]	< 0.0001	0	0	2.7 [2.1-3.5]	< 0.0001
**Transfer in neonatal department**	97 (100)	83 (100)		–	212 (53.0)	567 (39.8)	1.7 [1.4-2.1]	< 0.0001
**Infant respiratory distress syndrome**	22 (22.7)	18 (21.7)	1.07 [0.52-1.2]	0.87	2 (0.5)	3 (0.2)	2.4 [0.4-14]	0.32
**3-min Apgar score < 7**	29 (30.9)	27 (32.5)	0.93 [0.49-1.8]	0.81	59 (14.9)	90 (6.3)	2.6 [1.8-3.7]	< 0.0001
**Liveborns and fœtal deaths (malf excluded)**	**N = 120**	**N = 103**			**N = 409**	**N = 1428**		
**Outcome:** **• In utero fetal death** **• Medical termination** * death <28 days	17 (14.2) 6 (5.0) 11 (11.3)	16 (15.5) 4 (2.1) 4 (4.8)	0.90 1.3 2.5	0.77 0.69 0.11	9 (2.2) 0 0	3 (0.2) 0 2 (0.1)	10.7 [2.9-39] – –	< 0.0001 0.29

# recorded since 2005 in the database (instead of 2001).

Table 4 In the multivariable logistic regression analysis we compared PFGR to VFGR, across early-onset and late-onset subgroups. Several independent risk factors emerged with distinct patterns by timing (Table 4). For early-onset disease, chronic hypertension was the strongest predictor (adjusted OR 9.1, 95% CI 2.45–34, P = 0.001), followed by new father for the index pregnancy (adjusted OR 6.8, 95% CI 2.1–22, P = 0.001), previous abortion (adjusted OR 5.7, 95% CI 1.7–19, P = 0.005), and renal disease (adjusted OR 7.8, 95% CI 1.4–45, P = 0.02). Maternal age (per 5-year increment) also showed a robust association (adjusted OR 1.92, 95% CI 1.2–3.0, P = 0.005), while pre-pregnancy BMI (per 5 kg/m^2^ increment) was non-significant (adjusted OR 1.31, 95% CI 0.84–2.0, P = 0.23). Smoking, education level, previous preeclampsia, previous SGA, asthma, and thyroid disease were not independently associated (all P > 0.05). In contrast, for late-onset disease, new father (adjusted OR 2.1, 95% CI 1.5–3.1, P < 0.001) and chronic hypertension (adjusted OR 2.8, 95% CI 1.4–5.6, P = 0.003) retained significance but with attenuated effect sizes; maternal age (adjusted OR 1.4, 95% CI 1.2–1.7, P < 0.001) and BMI (adjusted OR 1.26, 95% CI 1.08–1.5, P = 0.003) were modestly associated, alongside previous abortion (adjusted OR 2.1, 95% CI 1.2–3.6, P = 0.01) and previous preeclampsia (adjusted OR 1.9, 95% CI 1.02–3.6, P = 0.04). Smoking was protective (adjusted OR 0.59, 95% CI 0.4–0.9, P = 0.008).

**Table 4:** Independent risk factors for PFGR versus VFGR according to gestational onset. § “extended primipaternity” (primigravid plus mutiparas with new partner). * multiparas.

RIK FACTOR PFGR (preeclamtic) vs VFGR (non Preeclamptic)	Early onset (< 34 weeks gestation) n = 223				Late onset (≥ 34 weeks gestation) n = 1837			
	Coeff.	OR	[95% CI]	p	Coeff.	OR	[95% CI]	p
**Maternal Age (increment of 5 years of age)**	0.65	**1.92**	[1.2-3.0]	0.005	0.34	**1.4**	[1.2-1.7]	< 0.001
**Pre-pregnancy BMI (increment of 5 kg/m²)**	0.27	**1.31**	[0.84-2.0]	0.23	−0.04	**1.26**	[1.08-1.5]	0.003
**Renal disease**	2.0	**7.8**	{1.4-45]	0.02	0.34	**1.4**		0.34
**Chronic hypertension**	2.2	**9.1**	[2.45-34]	0.001	0.23	**2.8**	[1.4-5.6]	0.003
**New father for the index pregnancy §**	1.21	**6.8**	[2.1-22]	0.001	1.08	**2.1**	[1.5-3.1]	< 0.001
**Smoking during pregnancy**	−0.49	**0.6**		0.43	−0.52	**0.59**	[0.4-0.9]	0.008
**10 years education (yes-no)**	−0.26	**0.77**		0.57	−0.27	**0.76**	[0.58-1.01]	0.06
Previous abortion *	1.7	**5.7**	[1.7-19]	0.005	0.73	**2.1**	[1.2-3.6]	0.01
Previous preeclampsia *	1.13	**2.9**	[0.7-11]	0.12	0.65	**1.9**	[1.02-3.6]	0.04

Fig 1 synthetizes our entire cohort of non-malformed SGA: Our results may be summarized in terms of quarters: Three quarters of SGA were “constitutional SGA”. The remaining quarter is obviously associated with “Doppler anomalies” detected during antenatal care, as surrogate markers of feto-placental insufficiency and as such FGR. Inside this minority of cases, three quarters of cases are VFGR, i.e., only the foetus is affected without any harm to the mother. Only one quarter of these FGR (PFGR) were associated with a harmful maternal disorder, preeclampsia, characterized by systemic endothelial dysfunction and inflammation. (proteinuria/glomeruloendotheliosis, HELLP, eclampsia..). It is noteworthy that the constitutional SGA curve is exactly similar to that of controls (women with normal pregnancy and without SGA at birth). Those constitutional SGA deliver exactly like controls, these newborns share exactly the same patterns except that they are of simply “smaller”. The horizontal bars displayed at the top of Fig 1 are schematic summaries of the observed frequencies of constitutional SGA, VFGR, and PFGR within the cohort and are not derived from a statistical model.

**Fig 2:**
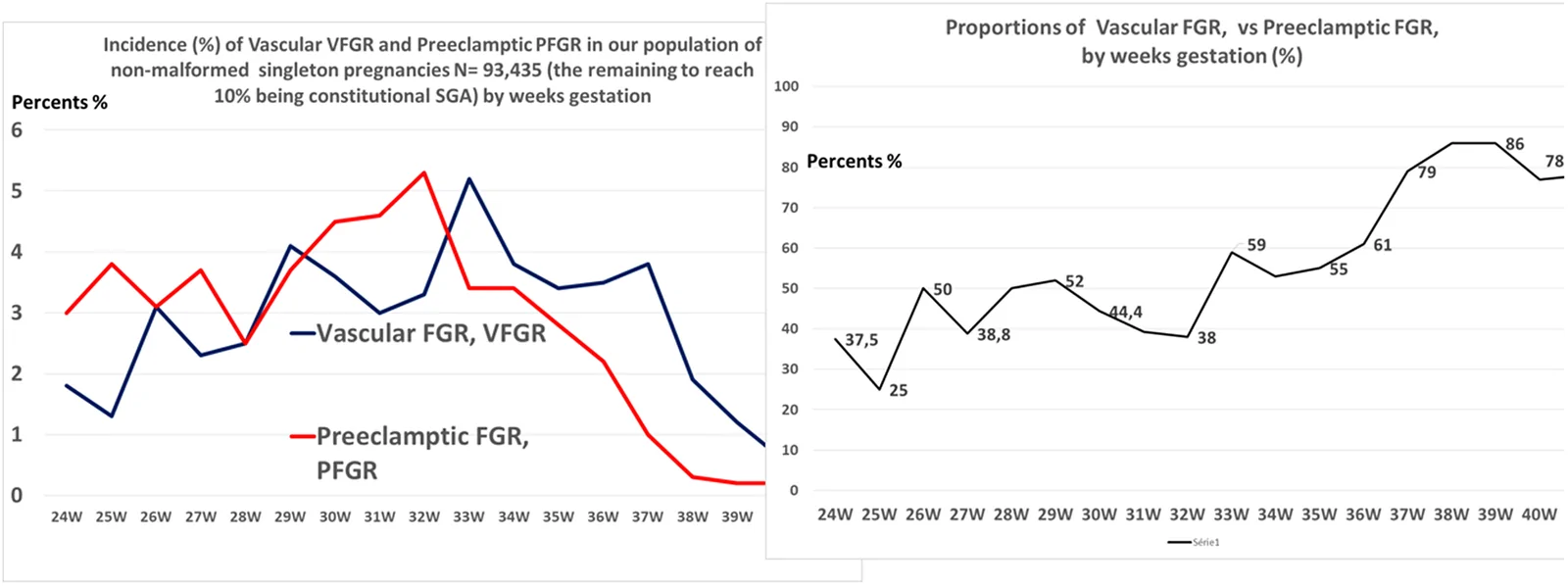
Distribution of our population of FGR foetuses/newborns, N = 2060 by weeks of gestation. Below 34 weeks N = 223, 34 weeks and after N = 1837.

Fig 2 shows the distribution of our population of FGR fetuses/newborns by date of deliveries (by weeks gestation). We can notice that in early deliveries below the 34^th^ week the populations of vascular and preeclamptic are similar (~ 50%)., with the same catastrophic outcomes, Table 3). It is after the 34^th^ week that VFGR become predominant, reaching more than 80% of cases near term.

## Discussion

This large population-based cohort study of 94,435 non-malformed singleton births over 24.5 years compared clinical risk factors between preeclampsia-associated fetal growth restriction (PFGR) and vascular fetal growth restriction without maternal preeclampsia (VFGR). By restricting FGR to SGA fetuses with clinically documented abnormal Doppler findings suggestive of placental insufficiency, we identified two distinct placental-mediated phenotypes: one associated with overt maternal disease (PFGR) and the other without maternal hypertensive manifestations (VFGR).

The relatively low prevalence of FGR observed in our cohort (2.2% of singleton pregnancies) is likely explained by our restrictive operational definition of “true” placental-mediated FGR. Only fetuses with suspected Doppler abnormalities during pregnancy follow-up were classified as FGR, whereas constitutionally small SGA newborns without evidence of placental vascular compromise were excluded from this category. In addition, malformed fetuses and congenital fetopathies were excluded. These methodological differences probably explain the lower prevalence compared with epidemiological studies using broader birthweight-based definitions of FGR. Our findings, to our own surprise, revealing that VFGR constitutes the majority (74.3%) of FGR cases, with PFGR accounting for only 25.7% [16], refine the classical view that maternal vascular malperfusion-associated FGR is predominantly linked to hypertensive maternal disease [8],

Although non-preeclamptic placental FGR has long been recognized [10–15], our findings emphasize that most Doppler-defined placental FGR cases occurred without clinically overt maternal preeclampsia, particularly in late-onset disease. Thus, while maternal vascular malperfusion remains central in the pathophysiology of placental insufficiency, our results suggest that maternal hypertensive disease represents only one possible maternal response to placental dysfunction rather than its obligatory clinical expression. This distinction was especially marked after 34 weeks of gestation, where VFGR largely predominated.

Key Clinical Distinctions and Their Implications. Several clinical characteristics differentiated PFGR from VFGR. PFGR mothers were older [17,18] and had higher pre-pregnancy BMI [19,20], whereas obesity and chronic hypertension were strongly associated with PFGR, particularly in early-onset disease. These findings are consistent with the concept that maternal metabolic and vascular vulnerability contributes to the development of systemic endothelial dysfunction in preeclampsia [19,20]. Conversely, underweight status and smoking were associated with lower rates of PFGR, consistent with the previously described “smoker’s paradox” in preeclampsia [21,22], although smoking remains a major risk factor for adverse perinatal outcomes overall and cannot obviously be considered protective in clinical practice.

Reproductive history also distinguished PFGR from VFGR. Previous preeclampsia and primipaternity were independently associated with PFGR, supporting the hypothesis that abnormal maternal immune tolerance to paternal-fetal antigens contributes to the maternal syndrome of preeclampsia [23–25]. These findings reinforce the concept that PFGR and VFGR may share placental insufficiency but differ in the maternal systemic response to placental stress signals.

Regarding neonatal outcomes, early-onset PFGR and VFGR were both associated with severe fetal and neonatal morbidity, suggesting that major placental dysfunction alone may lead to catastrophic fetal consequences irrespective of maternal hypertensive manifestations. In contrast, late-onset PFGR was associated with worse obstetrical and neonatal outcomes than late-onset VFGR, indicating an additive burden related to maternal disease.

### Orienting future biological research

Our findings pinpoint several susceptibility (e.g., obesity, advanced age, chronic hypertension, previous preeclampsia, primipaternity, renal disease, history of abortion) and protective (e.g., smoking, underweight, higher education, adolescence in late-onset) factors that distinguish PFGR from VFGR, offering a roadmap for genetic, epigenetic, and multi-omics investigations. These disparities suggest that PFGR may involve amplified maternal responses to placental signals, such as anti-angiogenic factors (e.g., sFlt-1) or inflammatory cytokines, that VFGR pregnancies tolerate without systemic spillover [26].

1. **Immunological Pathways**: The strong association with primipaternity implicates paternal-fetal antigen mismatch and deficient immune tolerance at the maternal-fetal interface [23,25]. Future studies should prioritize HLA genotyping, T-regulatory cell profiling, bisulfite DNA or single-cell RNA sequencing of decidual tissues to identify epigenetic modifiers (e.g., DNA methylation at immune loci) and molecular pathways that fail in PFGR but protect in VFGR [27].
2. **Metabolic and Vascular Endpoints**: Higher BMI, gestational weight gain, and chronic hypertension in PFGR point to dysregulated adipokines (e.g., leptin), insulin resistance, and eNOS polymorphisms [28]. Multi-omics approaches, including metabolomics (e.g., lipid profiles) and proteomics (e.g., vascular biomarkers like endoglin), could reveal why obesity exacerbates maternal syndrome in PFGR, potentially guiding trials of metformin or statins [29].
3. **Renal and Endocrine Axes**: Renal disease’s prominence in early-onset PFGR suggests podocyte injury and complement activation pathways. Integrated genomics (e.g., GWAS for preeclampsia susceptibility genes like FLT1) with epigenomics (e.g., histone modifications in renal endothelium) may uncover why some women progress to glomerular endotheliosis [27]. Thyroid associations warrant thyroid hormone receptor variant studies and autoimmune profiling [30].
4. **Reproductive History Markers**: The link to previous abortions hints at inherited thrombophilias (e.g., Factor V Leiden) or antiphospholipid syndromes [25]. Epigenetic analyses of endometrial samples could explore imprinting defects or miRNA dysregulation persisting across pregnancies.
5. **Protective Mechanisms**: Smoking’s paradoxical protection invites nicotine receptor (e.g., CHRNA7) and anti-inflammatory cytokine (e.g., IL-10) studies [22]. Underweight’s role may involve lower angiogenic imbalance, meriting nutrigenomics to identify caloric restriction mimetics.
6. **Timing-Specific Heterogeneity**: Early- vs. late-onset differences (e.g., stronger effects of chronic hypertension and renal disease in early-onset PFGR) support distinct etiologies: early-onset linked to defective placentation, late-onset to maternal maladaptation [31]. Longitudinal multi-omics cohorts, stratified by onset, could integrate placental histopathology with maternal blood omics to model these trajectories [27].

Our findings support the concept that PFGR and VFGR represent related but distinct phenotypes of placental insufficiency. The differential associations with obesity, chronic hypertension, primipaternity, previous preeclampsia, and smoking suggest that maternal susceptibility to systemic endothelial and inflammatory activation may determine whether placental dysfunction remains confined to the fetoplacental unit or progresses to overt maternal disease. These observations provide potential directions for future genetic, epigenetic, immunological, and multi-omics studies aimed at identifying the mechanisms that protect most women with placental insufficiency from developing preeclampsia.

## Conclusions

In this large population-based cohort, most Doppler-defined placental-mediated FGR cases occurred without maternal preeclampsia, particularly in late-onset disease. PFGR was associated with distinct maternal vascular, metabolic, and immunological risk factors compared with VFGR, supporting the concept that maternal susceptibility largely determines the development of overt preeclampsia in the setting of placental insufficiency. Better understanding of these protective and susceptibility mechanisms may improve future prediction, prevention, and management strategies for placental vascular diseases.

## Supporting information

S1 File Reunion neonatal curves. (DOCX)

## Abbreviations

SGA: small for gestational age (10th percentile)
Constitutional SGA: SGA without any doppler anomalies and without hypertensive disorders of pregnancies
FGR: fœtal growth retardation (doppler anomalies since the 2^nd^ trimester of gestation, stunted foetuses)
PFGR: fœtal growth retardation in a context of maternal preeclampsia
VFGR: fœtal growth retardation without maternal preeclampsia
