# Supplementary material for: Why only one-quarter of placental fetal growth restriction triggers preeclampsia? Clinical risk factors in 2060 cases in a 24-year cohort of 90,000 births
Source: PLoS One. 2026 Sep 18;21(9):e0354687. doi: 10.1371/journal.pone.0354687 (PMC13588347; doi:10.1371/journal.pone.0354687)
Supplement: Supplementary file 1 [file pone.0354687.s001.docx]

87 516 singleton pregnancies, fetal deaths and fœtal malformations excluded. PY Robillard CHU Sud-Réunion, 2022

**fœtal Growth**. **Réunion, 2022**

**N = 40 803 males , 39 985 females**

|  | **5thPercentile** | **5th**  **percentile** | **10th**  **percentile** | **10th**  **percentile** | **50th**  **percentile** | **50th**  **percentile** | **90th**  **percentile** | **90th**  **percentile** |
| --- | --- | --- | --- | --- | --- | --- | --- | --- |
| **SA** | **Male** | **Female** | **Male** | **Female** | **Male** | **Female** | **Male** | **Female** |
| **23** | 478 | 405 | **522** | **440** | 600 | 550 | **702** | **685** |
| **24** | 550 | 459 | **580** | **486** | 680 | 655 | **819** | **790** |
| **25** | 550 | 460 | **650** | **558** | 800 | 747 | **946** | **880** |
| **26** | 580 | 500 | **676** | **584** | 882 | 795 | **1052** | **1000** |
| **27** | 654 | 540 | **720** | **630** | 1000 | 890 | **1200** | **1130** |
| **28** | 746 | 640 | **790** | **720** | 1100 | 1010 | **1373** | **1258** |
| **29** | 885 | 704 | **910** | **820** | 1230 | 1160 | **1530** | **1430** |
| **30** | 970 | 815 | **993** | **890** | 1392 | 1280 | **1730** | **1673** |
| **31SA** | 1084 | 995 | **1191** | **1070** | 1600 | 1466 | **1918** | **1860** |
| **32** | 1233 | 1106 | **1359** | **1201** | 1765 | 1642 | **2162** | **2107** |
| **33** | 1356 | 1304 | **1530** | **1430** | 1970 | 1850 | **2370** | **2280** |
| **34** | 1569 | 1461 | **1740** | **1605** | 2240 | 2090 | **2670** | **2560** |
| **35 SA** | 1810 | 1710 | **1960** | **1859** | 2430 | 2340 | **2920** | **2860** |
| **36** | 2019 | 1922 | **2160** | **2060** | 2680 | 2560 | **3220** | **3125** |
| **37** | 2240 | 2120 | **2390** | **2280** | 2900 | 2800 | **3474** | **3350** |
| **38** | 2480 | 2360 | **2620** | **2500** | 3100 | 2980 | **3650** | **3520** |
| **39** | 2620 | 2540 | **2760** | **2670** | 3260 | 3130 | **3800** | **3650** |
| **40** | 2760 | 2650 | **2890** | **2780** | 3380 | 3250 | **3920** | **3780** |
| **41** | 2840 | 2730 | **2980** | **2880** | 3490 | 3340 | **4040** | **3890** |

**fœtal Growth**. **Réunion, 2022**

|  |  |  |  |  |
| --- | --- | --- | --- | --- |
| 22SA | 430 | 410 | 592 | 542 |
| 23SA | 522 | 440 | 702 | 685 |
| 24SA | 580 | 486 | 819 | 790 |
| 25SA | 650 | 558 | 946 | 880 |
| 26SA | 676 | 584 | 1052 | 1000 |
| 27SA | 720 | 630 | 1200 | 1130 |
| 28SA | 790 | 720 | 1373 | 1258 |
| 29SA | 910 | 820 | 1530 | 1430 |
| 30SA | 993 | 890 | 1730 | 1673 |
| 31SA | 1191 | 1070 | 1918 | 1860 |
| 32SA | 1359 | 1201 | 2162 | 2107 |
| 33SA | 1530 | 1430 | 2370 | 2280 |
| 34SA | 1740 | 1605 | 2670 | 2560 |
| 35SA | 1960 | 1859 | 2920 | 2860 |
| 36SA | 2160 | 2060 | 3220 | 3125 |
| 37SA | 2390 | 2280 | 3474 | 3350 |
| 38SA | 2620 | 2500 | 3650 | 3520 |
| 39SA | 2760 | 2670 | 3800 | 3650 |
| 40SA | 2890 | 2780 | 3920 | 3780 |
| 41SA | 2980 | 2880 | 4040 | 3890 |
