# Supplementary material for: Why only one-quarter of placental fetal growth restriction triggers preeclampsia? Clinical risk factors in 2060 cases in a 24-year cohort of 90,000 births
Source: PLoS One. 2026 Sep 18;21(9):e0354687. doi: 10.1371/journal.pone.0354687 (PMC13588347; doi:10.1371/journal.pone.0354687)
Supplement: Supplementary file 2 [file pone.0354687.s003.docx]

**Why Only One-Quarter of Placental Fetal Growth Restriction**

**Triggers Preeclampsia ? Clinical Risk Factors in 2060 Cases in a**

**24-Year Cohort of 90,000 births.**

We thank the reviewers for their important and fruitfull comments

**Reviewer #1:** General comments **MODIFCATIONS IN THE TEXT IN RED COLOUR**
This study is a retrospective observational study based on a prospectively collected hospital perinatal database covering all deliveries between 2001 and 2025. It aimed to describe differences in a priori defined risk factors between FGR related to preeclampsia and FGR not related to preeclampsia among non-malformed singleton births (N = 94,435). The findings are interesting; however, the interpretation that they challenge a traditional paradigm may be overstated.
Please carefully check the manuscript for typos and ensure consistency in the use of abbreviations.

1) **In the tables**, please present all odds ratios (ORs) with their confidence intervals, even when they are not statistically significant to being able to judge the magnitude of the associations. Please also round all numbers to the same number of decimal places, except for p-values.

**DONE**

2) **Introduction**
   The authors’ introduction mostly relies on their own previous research and lacks a broader general context.
   They state that “this observation directly challenges the classic textbook statement that the most common cause of FGR in a structurally normal fetus is maternal vascular malperfusion associated with preeclampsia.”supported by reference 10.1016/j.preghy.2010.10.013. However, this statement does not appear to be the primary focus of the cited article. The authors may wish to verify that this reference adequately supports the quoted assertion or provide a more appropriate source.

**Indeed, you are right, it is an incomprhensible mistake, the actual reference is Bamberg et al ref [8].** Bamberg C, Kalache KD. Prenatal diagnosis of fetal growth restriction. *Semin Fetal Neonatal Med*. 2004;9(5):387-394. doi:10.1016/j.siny.2004.03.007

Even in the absence of this incorrect reference, the authors state that their findings challenge the classic assertion that maternal vascular malperfusion associated with preeclampsia is the most common cause of FGR in structurally normal fetuses. However, maternal vascular malperfusion may occur in the absence of clinical preeclampsia. Therefore, the observation that only a minority of FGR cases were associated with preeclampsia does not necessarily contradict this paradigm unless placental pathology was systematically assessed.

We agree with the reviewer that maternal vascular malperfusion may occur in the absence of clinically overt preeclampsia, and that placental insufficiency is a well-recognized cause of isolated fetal growth restriction. Our intention was not to deny the role of placental malperfusion in FGR, but rather to highlight that, in our cohort, clinically diagnosed preeclampsia accounted for only a minority of FGR cases, consistent with previous reports showing that placental dysfunction and FGR frequently occur independently of maternal hypertensive manifestations. We have re-written the introduction, defining clearly the definitions of SGA and differences with FGR. Especially, the new references 9 to 13 cite studies showing that maternal vascular malperfusion may occur in the absence of preeclampsia

3) **Materials and Methods**
   Some abbreviations are introduced and later replaced by others, which is confusing when reading the manuscript (PES → PFGR and VascS → VFGR).

This has been corrected in methods

The manuscript indicates that FGR was defined based on Doppler abnormalities in both PFGR and VFGR. However, it appears that the diagnosis was cross-checked and confirmed using additional hospital records only for VFGR. Could the authors clarify whether a similar validation process was applied to PFGR cases? If confirmation procedures differed between groups, this could introduce differential diagnostic verification and potentially affect group comparisons.

Our diagnosis of FGR has been unfortunately indirect, as « Abnormal doppler conditions » does not exist as such in our database. This has been precised in a new « strengthst and limitations », page 18 (in blue, as it is also a demand by reviewer 2).

In the definitions of exposures and outcomes, several variables are not clearly defined.
How was SGA defined in this study? Which reference standards were used?

This has been precised in the new intoduction

How was smoking during pregnancy measured? It is unclear how this information was recorded in the hospital database.

This information does exist in the perinatal database as a qualitative item « yes/no » (direct inquiry with the mothers).

How was chronic hypertension defined? Was it directly reported in the medical record or identified through the use of antihypertensive medication? If medication use was used as a proxy, how were cases of chronic hypertension diagnosed during pregnancy before 20 weeks of gestation classified?

NO, antihypertensive drugs were not used as proxy. Chronic hypertension was defined as known hypertension before pregnancy and/or hypertension ≥140 mmhg (systolic)or ≥ 90 mmhg (diastolic) before the 20th week of gestation

The manuscript reports the occurrence of chronic hypertension among women with preeclampsia (YES, indeed). Could the authors clarify whether these cases correspond to superimposed preeclampsia? As chronic hypertension and preeclampsia are distinct diagnoses, further specification would improve clarity.
How was antihypertensive treatment integrated into the analysis?

Yes preeclampsia (proteinuria, HELLP) associated with chronic hypertension are superimposed preeclampsia. Noted in methods

How were data on paternity collected? Direct inquiry with the mother

How were data on maternal education level collected? Direct inquiry with the mother

**ALL THESE ITEMS HAVE BEEN PECISED IN METHODS**

4) **Results**
   The reported prevalence of FGR (~2%) appears lower than that reported in most epidemiological studies (https://pubmed.ncbi.nlm.nih.gov/33972065/). The authors should clarify whether differences in the operational definition of FGR may explain this discrepancy.

We thank the reviewer for this important comment. The apparently lower prevalence of FGR in our study is mainly explained by differences in the operational definition of FGR and by the strict exclusion criteria applied in our cohort.

First, we excluded all malformed fetuses and neonates (2.8% of singleton pregnancies), including congenital infections and fetal alcohol syndrome, conditions that are frequently included in epidemiological studies of SGA/FGR and that substantially contribute to the burden of growth restriction. After these exclusions, the prevalence of non-malformed SGA newborns was exactly 10% (9,356/94,435), consistent with the classical definition of SGA below the 10th percentile.

Second, unlike many epidemiological studies that classify FGR primarily on the basis of birthweight centiles, we deliberately distinguished constitutional SGA from true placenta-mediated growth restriction using prenatal Doppler evidence of impaired placental circulation

WE HAVE ADDED IN « DISCUSSION, page 16 this paragraph « The relatively low prevalence of FGR observed in our cohort (2.2% of singleton pregnancies) is likely explained by our restrictive operational definition of FGR. Only SGA fetuses with clinically documented suspected Doppler abnormalities during pregnancy follow-up were classified as FGR, including preeclampsia-associated FGR (PFGR, N=529) and vascular FGR without preeclampsia (VFGR, N=1,531). In contrast, constitutionally small SGA newborns without evidence of placental vascular compromise were not considered growth restricted. In addition, all malformed fetuses and congenital foetopathies were excluded from the analysis. These methodological differences probably account for the lower prevalence observed in our study compared with population-based epidemiological studies using broader definitions of FGR primarily based on birthweight centiles. »

Please rephrase “To be adolescent” as “Being younger than 18 years” or “Adolescent age (<18 years)”. **CORRECTED**

Since gestational weight gain occurs concomitantly with preeclampsia and may be influenced by its diagnosis, caution is warranted in interpreting this association due to potential reverse causality.

You are right, edema in PE could influence GWG. We have deleted this line in Table 1 (and comments in results)

For the sake of consistency, please provide a proper title for the regression model results rather than “Table 4”. The same applies to “Figure 1”, or alternatively place these references in parentheses when referring to them in the text.

We thank the reviewer for this helpful suggestion. We have revised the manuscript to provide explicit descriptive titles for the regression model results and figures instead of referring to them generically as “Table 4” or “Figure 1”. In addition, figure and table references in the text have been standardized and, where appropriate, placed in parentheses for consistency with journal style.

The authors report an association that does not appear in the results (and therefore should not be presented as part of the current analysis:
“constitutional SGA rather linked with low maternal BMI and ethnicity.”

This sentence has been removed from the text

It is not clear how the horizontal line representing the proportions of different SGA categories at the top of Figure 1 was produced. Please clarify.

We thank the reviewer for pointing out this lack of clarity. The horizontal line displayed at the top of Figure 1 is a schematic representation summarizing the proportions of the different SGA categories within the entire cohort. It was constructed directly from the observed frequencies in the study population: approximately 75% constitutional SGA, 25% FGR among all SGA cases, and within FGR, approximately 75% VFGR and 25% PFGR. We have clarified this point in the revised legend of Figure 1 and in the Methods/Results section to indicate that these proportions are descriptive summaries derived from the cohort data rather than outputs from a statistical model.

Please rephrase the sentence:
“Only one quarter of these ‘poor Doppler pregnancies’, PFGR? are associated…”

It has been replaced by « Only one quarter of these FGR (PFGR) were associated with a harmful maternal disorder, preeclampsia, characterized by systemic endothelial dysfunction and inflammation » page 15

5) **Discussion**
   The authors report that only one quarter of FGR cases were associated with preeclampsia and interpret this finding as challenging the “traditional paradigm that preeclampsia is the predominant driver of FGR.” However, FGR is generally recognized as a multifactorial condition, and many cases occur in the absence of maternal preeclampsia, particularly in late-onset disease.

The distribution observed here therefore appears broadly consistent with current understanding. The authors should clarify which specific paradigm or prior evidence is being challenged by their findings.

We thank the reviewer for this important comment and agree that fetal growth restriction (FGR) is currently recognized as a multifactorial condition, with many cases occurring in the absence of maternal preeclampsia, particularly in late-onset disease. Our intention was not to suggest that non-preeclamptic FGR is a novel concept, which has indeed been previously described in the literature and incorporated into current classifications of placental insufficiency and late-onset FGR.

We have therefore revised the Discussion to clarify that the paradigm being challenged is the persistent classical view that placental vascular malperfusion leading to FGR is predominantly linked to maternal preeclampsia or hypertensive disease. This concept is still reflected in many reviews and textbooks describing maternal vascular malperfusion as the principal mechanism underlying FGR in structurally normal fetuses.

Our findings do not contradict the multifactorial nature of FGR, but rather emphasize quantitatively that in a large Doppler-defined placental FGR population, the majority of cases (74%) occurred without clinically overt maternal preeclampsia, especially after 34 weeks’ gestation. We therefore believe that the key issue raised by our data is not the existence of non-preeclamptic FGR itself, but the relative predominance of maternal “tolerance” phenotypes (VFGR) over maternal systemic disease phenotypes (PFGR), despite apparently similar placental vascular insufficiency.

The manuscript has been revised accordingly to temper the wording and better distinguish between historical concepts of “maternal vascular malperfusion-associated FGR” and the contemporary understanding of heterogeneous placental FGR phenotypes.

WE HAVE ADDED A PARAGRAPH page 16.

**Reviewer #2:** In this cohort study attention was focused on placenta-related FGR, associated or not to Preeclampsia/eclampsia.

The large cohort suffers of time spanning. Neonatology markedly improved along years with outcome not simply comparable. Moreover, the definition of FGR are not granted to be obtained with same parameter and could allow heterogeneity during such a long period of data collection.

1. Being a single-center study, data collection took over 23 yrs period. Meanwhile, definition of placenta FGR have been changed. How the Authors accommodate their labelling according the to the last Delphi consensus.

**We thank the reviewer for this important methodological comment.**

**First, regarding temporal changes in neonatal management, we acknowledge that obstetrical and neonatal practices evolved during the 24.5-year study period. However, the principal objective of the present study was not to compare neonatal survival across eras, but rather to compare maternal clinical characteristics and risk-factor profiles between preeclampsia-associated FGR (PFGR) and non-preeclamptic vascular FGR (VFGR). These maternal phenotypic associations are less likely to be substantially influenced by improvements in neonatal intensive care over time.**

**Second, we fully agree that definitions of placental fetal growth restriction (FGR) evolved during the long study period, particularly with the publication of the international Delphi consensus criteria for FGR diagnosis (our revised reference 14). However, our study was not designed as a retrospective reclassification of all pregnancies according to contemporary Delphi criteria. Instead, it reflects a real-world prospective clinical cohort in which cases labelled as “FGR” corresponded to pregnancies recognized and managed by obstetricians as placental vascular FGR during routine care.**

**Third, importantly, the study database started in January 2001, at a period when Doppler velocimetry had already become integrated into standard obstetrical evaluation of suspected placental insufficiency following the seminal studies of the late 1980s and 1990s. Throughout the study period, the diagnosis of FGR in our center relied on abnormal fetal growth trajectories associated with Doppler abnormalities and clinical surveillance for placental insufficiency, which is conceptually consistent with the pathophysiological framework later formalized by the Delphi consensus.**

**The Delphi procedure mainly standardized and refined diagnostic thresholds and combinations of biometric and Doppler criteria, particularly for early- versus late-onset FGR, rather than introducing an entirely new disease entity. Therefore, although exact Delphi variables were not prospectively coded in our database throughout the entire 24.5-year period, the pregnancies identified as VFGR or PFGR correspond clinically to what would currently be considered placenta-mediated Doppler-defined FGR. Importantly, FGR cases were not identified retrospectively from birthweight centiles alone, but from prospectively coded clinical indications recorded by physicians or midwives (labor induction, cesarean delivery, hospitalization in high-risk pregnancy units, prenatal diagnosis, etc.), thereby reflecting real-life clinical recognition of placental vascular FGR.**

**To address this issue transparently, we have added a paragraph in the “Strengths and limitations” section acknowledging that our operational definition of FGR relied on prospectively recorded clinical diagnoses and management indications rather than retrospective application of Delphi criteria to all cases. We also emphasize that the internal coherence of the cohort strongly supports the validity of this classification, as constitutional SGA pregnancies behaved similarly to non-SGA controls, whereas VFGR and PFGR displayed clearly distinct maternal and neonatal profiles (see Figure 1).**

**Finally, we believe that the internal consistency of the cohort supports the validity of this classification. As illustrated in Figure 1, constitutional SGA pregnancies displayed gestational-age distributions and epidemiological profiles remarkably similar to normotensive non-SGA controls, whereas VFGR and PFGR demonstrated clearly distinct clinical patterns and outcomes (ref 16, our first study on the subject). These findings support the robustness of the operational distinction used in the study.**

Hypothesis raised in the paper is however stimulating.


2. Page 3: controls included birthweight till 100th centile (according to sex and gestational age). This is questionable since perinatology papers define Large for gestational age newborns above 90th or 97th centile suggesting a growth disorders. A vast literature is devoted to them. In my mind controls should remain in the range 10 to 90th centile.

**We thank the reviewer for this thoughtful comment.**

**We agree that large-for-gestational-age (LGA) newborns, commonly defined above the 90th or 97th percentile, represent a distinct fetal growth phenotype associated with specific maternal and neonatal risks. However, the objective of the present study was to compare different subtypes of small-for-gestational-age (SGA) and fetal growth restriction (FGR) pregnancies against the overall background population of normotensive non-SGA pregnancies.**

**For this reason, our control group was defined pragmatically as all non-malformed singleton pregnancies without SGA and without hypertensive disorders of pregnancy. We acknowledge that this definition therefore includes a minority of LGA infants. However, given the relatively limited proportion of LGA newborns within the total cohort, we believe their inclusion is unlikely to have materially influenced the observed associations between PFGR and VFGR, particularly as the principal analyses focused on comparisons within FGR phenotypes rather than on direct contrasts with controls.**

**Importantly, the study was not designed to investigate fetal overgrowth disorders or metabolic phenotypes associated with LGA pregnancies. Restricting controls retrospectively to the 10th–90th percentile range would require complete reconstruction of all comparative analyses across the entire 24.5-year cohort, without likely altering the major conclusions of the study.**

**Nevertheless, we agree that inclusion of LGA pregnancies in the control population may represent a methodological limitation, and we have now acknowledged this point in the revised manuscript.**

3. Table 3 (and related text): The terminology of “term gestation” is confounding, replace with gestational age; term gestation < 37 weeks should be replaced by Preterm birth <37, < 33. **CORRECTED**

Moreover, there should be something wrong in the OR of 3-min Apgar score <7.

Thank you, you are right, corrected 0.93 [0.49-1.8]

4. Table 4. The heading is not “Outcome…” replace with “Risk Factor”. **CORRECTED**

In several columns and in the entire last 3 lines reported risk factors are NOT statistically significant at the multivariate analysis: remove them from the table.

**THE THREE LAST COLUMNS HAVE BEEN DELETED**

5. Overall, non-significant different comparisons cannot be associated to an OR and related CI: please, delete from every line, in every table.

We thank the reviewer for this comment.

We agree that non-significant associations should be interpreted cautiously. However, we elected to retain odds ratios and their 95% confidence intervals for all comparisons, including non-significant results, in accordance with standard epidemiological reporting practices. Reporting effect sizes together with confidence intervals allows readers to assess both the magnitude and precision of the observed associations, independently of statistical significance alone.

This approach was also requested by another reviewer and is consistent with current recommendations emphasizing estimation rather than dichotomous interpretation based solely on p-values.

**NB** REVIEWER 1 : « **In the tables**, please present all odds ratios (ORs) with their confidence intervals, even when they are not statistically significant to being able to judge the magnitude of the associations. Please also round all numbers to the same number of decimal places, except for p-values »

6. Discussion at page 16 and 17 should be condensed, avoiding numbers already reported in results/tables. Meanwhile, hypotheses raised in at page 18 should be part of that reasoning. At any case, “trends” as well as non-significant changes cannot be part of those reasoning. They are overinterpretations and should be deleted from the paper.

**We thank the reviewer for this important comment. We have substantially condensed and reorganized the Discussion section. Numerical results already detailed in the Results section and Tables have been reduced to avoid redundancy, and the mechanistic/evolutionary hypotheses previously developed later in the Discussion have now been integrated into the corresponding thematic paragraphs to improve readability and coherence.**

**In addition, we removed references to “trends” or non-significant associations and restricted the interpretation to statistically supported findings only, in order to avoid overinterpretation.**

7. Please refer which ethnic group pertains to the population enrolled.

**French regulations governing medical and epidemiological databases do not permit the routine collection of data on ethnicity, religion, or political affiliation in perinatal registries. Consequently, ethnicity was not recorded in our database and could not be analyzed in the present study.**
