# Supplementary material for: Why only one-quarter of placental fetal growth restriction triggers preeclampsia? Clinical risk factors in 2060 cases in a 24-year cohort of 90,000 births
Source: PLoS One. 2026 Sep 18;21(9):e0354687. doi: 10.1371/journal.pone.0354687 (PMC13588347; doi:10.1371/journal.pone.0354687)
Supplement: Peer review history

# Peer review: Decision Letter 0

**DOI:** 10.1371/journal.pone.0354687.r001

**Authors:** Umberto Simeoni

12 Apr 2026

Triggers Preeclampsia ? Clinical Risk Factors in 2060 Cases in a

Dear Dr. Robillard,

Please submit your revised manuscript by May 27 2026 11:59PM. If you will need more time than this to complete your revisions, please reply to this message or contact the journal office at plosone@plos.org. When you're ready to submit your revision, log on to https://www.editorialmanager.com/pone/ and select the 'Submissions Needing Revision' folder to locate your manuscript file.

- A letter that responds to each point raised by the academic editor and reviewer(s). You should upload this letter as a separate file labeled 'Response to Reviewers'.
- A marked-up copy of your manuscript that highlights changes made to the original version. You should upload this as a separate file labeled 'Revised Manuscript with Track Changes'.
- An unmarked version of your revised paper without tracked changes. You should upload this as a separate file labeled 'Manuscript'.

If you would like to make changes to your financial disclosure, please include your updated statement in your cover letter. Guidelines for resubmitting your figure files are available below the reviewer comments at the end of this letter.

If applicable, we recommend that you deposit your laboratory protocols in protocols.io to enhance the reproducibility of your results. Protocols.io assigns your protocol its own identifier (DOI) so that it can be cited independently in the future. For instructions see: https://journals.plos.org/plosone/s/submission-guidelines#loc-laboratory-protocols. Additionally, PLOS ONE offers an option for publishing peer-reviewed Lab Protocol articles, which describe protocols hosted on protocols.io. Read more information on sharing protocols at https://plos.org/protocols?utm_medium=editorial-email&utm_source=authorletters&utm_campaign=protocols.

As the corresponding author, your ORCID iD is verified in the submission system and will appear in the published article. PLOS supports the use of ORCID, and we encourage all coauthors to register for an ORCID iD and use it as well. Please encourage your coauthors to verify their ORCID iD within the submission system before final acceptance, as unverified ORCID iDs will not appear in the published article. *Only*  the individual author can complete the verification step; PLOS staff *cannot*  verify ORCID iDs on behalf of authors.

We look forward to receiving your revised manuscript.

Kind regards,

Umberto Simeoni

Academic Editor

PLOS One

Journal Requirements:

When submitting your revision, we need you to address these additional requirements.

1.Please ensure that your manuscript meets PLOS ONE's style requirements, including those for file naming. The PLOS ONE style templates can be found at

https://journals.plos.org/plosone/s/file?id=wjVg/PLOSOne_formatting_sample_main_body.pdf and

https://journals.plos.org/plosone/s/file?id=ba62/PLOSOne_formatting_sample_title_authors_affiliations.pdf

2. Please include captions for your Supporting Information files at the end of your manuscript, and update any in-text citations to match accordingly. Please see our Supporting Information guidelines for more information: http://journals.plos.org/plosone/s/supporting-information.

3. Please note that your Data Availability Statement is currently missing the repository name and/or the DOI/accession number of each dataset OR a direct link to access each database. If your manuscript is accepted for publication, you will be asked to provide these details on a very short timeline. We therefore suggest that you provide this information now, though we will not hold up the peer review process if you are unable.

4. If the reviewer comments include a recommendation to cite specific previously published works, please review and evaluate these publications to determine whether they are relevant and should be cited. There is no requirement to cite these works unless the editor has indicated otherwise.

Additional Editor Comments:

Despite its interest, the study reported in the manuscript, as well as the interpretation of the findings have numerous limitations. They have been precisely detailed by the reviewers in their respective reports. In addition, the title of the manuscript is questionable, as, while preeclampsia has origins in the placenta, it is unknown whether it is triggered by fetal growth restriction, or whether fetal growth restriction is triggered by the placental changes related to preeclampsia.

Whether all these limitations can be addressed in a revised version of the manuscript is uncertain. However the authors are invited to submit a revised version of the manuscript, that addresses all the comments made by the reviewers.

[Note: HTML markup is below. Please do not edit.]

Reviewer's Responses to Questions

**Comments to the Author**

1. Is the manuscript technically sound, and do the data support the conclusions?

Reviewer #1: Partly

Reviewer #2: Partly

**********

2. Has the statistical analysis been performed appropriately and rigorously?

Reviewer #1: Yes

Reviewer #2: I Don't Know

**********

3. Have the authors made all data underlying the findings in their manuscript fully available?

The PLOS Data policy

Reviewer #1: Yes

Reviewer #2: Yes

**********

4. Is the manuscript presented in an intelligible fashion and written in standard English?

Reviewer #1: No

Reviewer #2: Yes

**********

Reviewer #1: General comments

This study is a retrospective observational study based on a prospectively collected hospital perinatal database covering all deliveries between 2001 and 2025. It aimed to describe differences in a priori defined risk factors between FGR related to preeclampsia and FGR not related to preeclampsia among non-malformed singleton births (N = 94,435). The findings are interesting; however, the interpretation that they challenge a traditional paradigm may be overstated.

Please carefully check the manuscript for typos and ensure consistency in the use of abbreviations.

In the tables, please present all odds ratios (ORs) with their confidence intervals, even when they are not statistically significant to being able to judge the magnitude of the associations. Please also round all numbers to the same number of decimal places, except for p-values.

Introduction

The authors’ introduction mostly relies on their own previous research and lacks a broader general context.

They state that “this observation directly challenges the classic textbook statement that the most common cause of FGR in a structurally normal fetus is maternal vascular malperfusion associated with preeclampsia.”supported by reference 10.1016/j.preghy.2010.10.013. However, this statement does not appear to be the primary focus of the cited article. The authors may wish to verify that this reference adequately supports the quoted assertion or provide a more appropriate source.

Even in the absence of this incorrect reference, the authors state that their findings challenge the classic assertion that maternal vascular malperfusion associated with preeclampsia is the most common cause of FGR in structurally normal fetuses. However, maternal vascular malperfusion may occur in the absence of clinical preeclampsia. Therefore, the observation that only a minority of FGR cases were associated with preeclampsia does not necessarily contradict this paradigm unless placental pathology was systematically assessed.

Materials and Methods

Some abbreviations are introduced and later replaced by others, which is confusing when reading the manuscript (PES → PFGR and VascS → VFGR).

The manuscript indicates that FGR was defined based on Doppler abnormalities in both PFGR and VFGR. However, it appears that the diagnosis was cross-checked and confirmed using additional hospital records only for VFGR. Could the authors clarify whether a similar validation process was applied to PFGR cases? If confirmation procedures differed between groups, this could introduce differential diagnostic verification and potentially affect group comparisons.

In the definitions of exposures and outcomes, several variables are not clearly defined.

How was SGA defined in this study? Which reference standards were used?

How was smoking during pregnancy measured? It is unclear how this information was recorded in the hospital database.

How was chronic hypertension defined? Was it directly reported in the medical record or identified through the use of antihypertensive medication? If medication use was used as a proxy, how were cases of chronic hypertension diagnosed during pregnancy before 20 weeks of gestation classified?

The manuscript reports the occurrence of chronic hypertension among women with preeclampsia. Could the authors clarify whether these cases correspond to superimposed preeclampsia? As chronic hypertension and preeclampsia are distinct diagnoses, further specification would improve clarity.

How was antihypertensive treatment integrated into the analysis?

How were data on paternity collected?

How were data on maternal education level collected?

Results

The reported prevalence of FGR (~2%) appears lower than that reported in most epidemiological studies (https://pubmed.ncbi.nlm.nih.gov/33972065/). The authors should clarify whether differences in the operational definition of FGR may explain this discrepancy.

Please rephrase “To be adolescent” as “Being younger than 18 years” or “Adolescent age (<18 years)”.

Since gestational weight gain occurs concomitantly with preeclampsia and may be influenced by its diagnosis, caution is warranted in interpreting this association due to potential reverse causality.

For the sake of consistency, please provide a proper title for the regression model results rather than “Table 4”. The same applies to “Figure 1”, or alternatively place these references in parentheses when referring to them in the text.

The authors report an association that does not appear in the results (and therefore should not be presented as part of the current analysis:

“constitutional SGA rather linked with low maternal BMI and ethnicity.”

It is not clear how the horizontal line representing the proportions of different SGA categories at the top of Figure 1 was produced. Please clarify.

Please rephrase the sentence:

“Only one quarter of these ‘poor Doppler pregnancies’, PFGR? are associated…”

Discussion

The authors report that only one quarter of FGR cases were associated with preeclampsia and interpret this finding as challenging the “traditional paradigm that preeclampsia is the predominant driver of FGR.” However, FGR is generally recognized as a multifactorial condition, and many cases occur in the absence of maternal preeclampsia, particularly in late-onset disease.

The distribution observed here therefore appears broadly consistent with current understanding. The authors should clarify which specific paradigm or prior evidence is being challenged by their findings.

Reviewer #2: In this cohort study attention was focused on placenta-related FGR, associated or not to Preeclampsia/eclampsia.

The large cohort suffers of time spanning. Neonatology markedly improved along years with outcome not simply comparable. Moreover, the definition of FGR are not granted to be obtained with same parameter and could allow heterogeneity during such a long period of data collection.

Hypothesis raised in the paper is however stimulating.

1. Being a single-center study, data collection took over 23 yrs period. Meanwhile, definition of placenta FGR have been changed. How the Authors accommodate their labelling according the to the last Delphi consensus.

2. Page 3: controls included birthweight till 100th centile (according to sex and gestational age). This is questionable since perinatology papers define Large for gestational age newborns above 90th or 97th centile suggesting a growth disorders. A vast literature is devoted to them. In my mind controls should remain in the range 10 to 90th centile.

3. Table 3 (and related text): The terminology of “term gestation” is confounding, replace with gestational age; term gestation < 37 weeks should be replaced by Preterm birth <37, < 33. Moreover, there should be something wrong in the OR of 3-min Apgar score <7.

4. Table 4. The heading is not “Outcome…” replace with “Risk Factor”. In several columns and in the entire last 3 lines reported risk factors are NOT statistically significant at the multivariate analysis: remove them from the table.

5. Overall, non-significant different comparisons cannot be associated to an OR and related CI: please, delete from every line, in every table.

6. Discussion at page 16 and 17 should be condensed, avoiding numbers already reported in results/tables. Meanwhile, hypotheses raised in at page 18 should be part of that reasoning. At any case, “trends” as well as non-significant changes cannot be part of those reasoning. They are overinterpretations and should be deleted from the paper.

7. Please refer which ethnic group pertains to the population enrolled.

**********

what does this mean?). If published, this will include your full peer review and any attached files.

If you choose “no”, your identity will remain anonymous but your review may still be made public.

**Do you want your identity to be public for this peer review?**  For information about this choice, including consent withdrawal, please see our Privacy Policy

Reviewer #1: No

Reviewer #2: No

**********

[NOTE: If reviewer comments were submitted as an attachment file, they will be attached to this email and accessible via the submission site. Please log into your account, locate the manuscript record, and check for the action link "View Attachments". If this link does not appear, there are no attachment files.]

To ensure your figures meet our technical requirements, please review our figure guidelines: https://journals.plos.org/plosone/s/figures

You may also use PLOS’s free figure tool, NAAS, to help you prepare publication quality figures: https://journals.plos.org/plosone/s/figures#loc-tools-for-figure-preparation.

NAAS will assess whether your figures meet our technical requirements by comparing each figure against our figure specifications.

# Author comment: Author response to Decision Letter 1

**DOI:** 10.1371/journal.pone.0354687.r002

16 Jun 2026

Why Only One-Quarter of Placental Fetal Growth Restriction

Triggers Preeclampsia ? Clinical Risk Factors in 2060 Cases in a

24-Year Cohort of 90,000 births.

We thank the reviewers for their important and fruitfull comments

Reviewer #1: General comments MODIFCATIONS IN THE TEXT IN RED COLOUR

This study is a retrospective observational study based on a prospectively collected hospital perinatal database covering all deliveries between 2001 and 2025. It aimed to describe differences in a priori defined risk factors between FGR related to preeclampsia and FGR not related to preeclampsia among non-malformed singleton births (N = 94,435). The findings are interesting; however, the interpretation that they challenge a traditional paradigm may be overstated.

Please carefully check the manuscript for typos and ensure consistency in the use of abbreviations.

1) In the tables, please present all odds ratios (ORs) with their confidence intervals, even when they are not statistically significant to being able to judge the magnitude of the associations. Please also round all numbers to the same number of decimal places, except for p-values.

DONE

2) Introduction

The authors’ introduction mostly relies on their own previous research and lacks a broader general context.

They state that “this observation directly challenges the classic textbook statement that the most common cause of FGR in a structurally normal fetus is maternal vascular malperfusion associated with preeclampsia.”supported by reference 10.1016/j.preghy.2010.10.013. However, this statement does not appear to be the primary focus of the cited article. The authors may wish to verify that this reference adequately supports the quoted assertion or provide a more appropriate source.

Indeed, you are right, it is an incomprhensible mistake, the actual reference is Bamberg et al ref [8]. Bamberg C, Kalache KD. Prenatal diagnosis of fetal growth restriction. Semin Fetal Neonatal Med. 2004;9(5):387-394. doi:10.1016/j.siny.2004.03.007

Even in the absence of this incorrect reference, the authors state that their findings challenge the classic assertion that maternal vascular malperfusion associated with preeclampsia is the most common cause of FGR in structurally normal fetuses. However, maternal vascular malperfusion may occur in the absence of clinical preeclampsia. Therefore, the observation that only a minority of FGR cases were associated with preeclampsia does not necessarily contradict this paradigm unless placental pathology was systematically assessed.

We agree with the reviewer that maternal vascular malperfusion may occur in the absence of clinically overt preeclampsia, and that placental insufficiency is a well-recognized cause of isolated fetal growth restriction. Our intention was not to deny the role of placental malperfusion in FGR, but rather to highlight that, in our cohort, clinically diagnosed preeclampsia accounted for only a minority of FGR cases, consistent with previous reports showing that placental dysfunction and FGR frequently occur independently of maternal hypertensive manifestations. We have re-written the introduction, defining clearly the definitions of SGA and differences with FGR. Especially, the new references 9 to 13 cite studies showing that maternal vascular malperfusion may occur in the absence of preeclampsia

3) Materials and Methods

Some abbreviations are introduced and later replaced by others, which is confusing when reading the manuscript (PES → PFGR and VascS → VFGR).

This has been corrected in methods

The manuscript indicates that FGR was defined based on Doppler abnormalities in both PFGR and VFGR. However, it appears that the diagnosis was cross-checked and confirmed using additional hospital records only for VFGR. Could the authors clarify whether a similar validation process was applied to PFGR cases? If confirmation procedures differed between groups, this could introduce differential diagnostic verification and potentially affect group comparisons.

Our diagnosis of FGR has been unfortunately indirect, as « Abnormal doppler conditions » does not exist as such in our database. This has been precised in a new « strengthst and limitations », page 18 (in blue, as it is also a demand by reviewer 2).

In the definitions of exposures and outcomes, several variables are not clearly defined.

How was SGA defined in this study? Which reference standards were used?

This has been precised in the new intoduction

How was smoking during pregnancy measured? It is unclear how this information was recorded in the hospital database.

This information does exist in the perinatal database as a qualitative item « yes/no » (direct inquiry with the mothers).

How was chronic hypertension defined? Was it directly reported in the medical record or identified through the use of antihypertensive medication? If medication use was used as a proxy, how were cases of chronic hypertension diagnosed during pregnancy before 20 weeks of gestation classified?

NO, antihypertensive drugs were not used as proxy. Chronic hypertension was defined as known hypertension before pregnancy and/or hypertension ≥140 mmhg (systolic)or ≥ 90 mmhg (diastolic) before the 20th week of gestation

The manuscript reports the occurrence of chronic hypertension among women with preeclampsia (YES, indeed). Could the authors clarify whether these cases correspond to superimposed preeclampsia? As chronic hypertension and preeclampsia are distinct diagnoses, further specification would improve clarity.

How was antihypertensive treatment integrated into the analysis?

Yes preeclampsia (proteinuria, HELLP) associated with chronic hypertension are superimposed preeclampsia. Noted in methods

How were data on paternity collected? Direct inquiry with the mother

How were data on maternal education level collected? Direct inquiry with the mother

ALL THESE ITEMS HAVE BEEN PECISED IN METHODS

4) Results

The reported prevalence of FGR (~2%) appears lower than that reported in most epidemiological studies (https://pubmed.ncbi.nlm.nih.gov/33972065/). The authors should clarify whether differences in the operational definition of FGR may explain this discrepancy.

We thank the reviewer for this important comment. The apparently lower prevalence of FGR in our study is mainly explained by differences in the operational definition of FGR and by the strict exclusion criteria applied in our cohort.

First, we excluded all malformed fetuses and neonates (2.8% of singleton pregnancies), including congenital infections and fetal alcohol syndrome, conditions that are frequently included in epidemiological studies of SGA/FGR and that substantially contribute to the burden of growth restriction. After these exclusions, the prevalence of non-malformed SGA newborns was exactly 10% (9,356/94,435), consistent with the classical definition of SGA below the 10th percentile.

Second, unlike many epidemiological studies that classify FGR primarily on the basis of birthweight centiles, we deliberately distinguished constitutional SGA from true placenta-mediated growth restriction using prenatal Doppler evidence of impaired placental circulation

WE HAVE ADDED IN « DISCUSSION, page 16 this paragraph « The relatively low prevalence of FGR observed in our cohort (2.2% of singleton pregnancies) is likely explained by our restrictive operational definition of FGR. Only SGA fetuses with clinically documented suspected Doppler abnormalities during pregnancy follow-up were classified as FGR, including preeclampsia-associated FGR (PFGR, N=529) and vascular FGR without preeclampsia (VFGR, N=1,531). In contrast, constitutionally small SGA newborns without evidence of placental vascular compromise were not considered growth restricted. In addition, all malformed fetuses and congenital foetopathies were excluded from the analysis. These methodological differences probably account for the lower prevalence observed in our study compared with population-based epidemiological studies using broader definitions of FGR primarily based on birthweight centiles. »

Please rephrase “To be adolescent” as “Being younger than 18 years” or “Adolescent age (<18 years)”. CORRECTED

Since gestational weight gain occurs concomitantly with preeclampsia and may be influenced by its diagnosis, caution is warranted in interpreting this association due to potential reverse causality.

You are right, edema in PE could influence GWG. We have deleted this line in Table 1 (and comments in results)

For the sake of consistency, please provide a proper title for the regression model results rather than “Table 4”. The same applies to “Figure 1”, or alternatively place these references in parentheses when referring to them in the text.

We thank the reviewer for this helpful suggestion. We have revised the manuscript to provide explicit descriptive titles for the regression model results and figures instead of referring to them generically as “Table 4” or “Figure 1”. In addition, figure and table references in the text have been standardized and, where appropriate, placed in parentheses for consistency with journal style.

The authors report an association that does not appear in the results (and therefore should not be presented as part of the current analysis:

“constitutional SGA rather linked with low maternal BMI and ethnicity.”

This sentence has been removed from the text

It is not clear how the horizontal line representing the proportions of different SGA categories at the top of Figure 1 was produced. Please clarify.

We thank the reviewer for pointing out this lack of clarity. The horizontal line displayed at the top of Figure 1 is a schematic representation summarizing the proportions of the different SGA categories within the entire cohort. It was constructed directly from the observed frequencies in the study population: approximately 75% constitutional SGA, 25% FGR among all SGA cases, and within FGR, approximately 75% VFGR and 25% PFGR. We have clarified this point in the revised legend of Figure 1 and in the Methods/Results section to indicate that these proportions are descriptive summaries derived from the cohort data rather than outputs from a statistical model.

Please rephrase the sentence:

“Only one quarter of these ‘poor Doppler pregnancies’, PFGR? are associated…”

It has been replaced by « Only one quarter of these FGR (PFGR) were associated with a harmful maternal disorder, preeclampsia, characterized by systemic endothelial dysfunction and inflammation » page 15

5) Discussion

The authors report that only one quarter of FGR cases were associated with preeclampsia and interpret this finding as challenging the “traditional paradigm that preeclampsia is the predominant driver of FGR.” However, FGR is generally recognized as a multifactorial condition, and many cases occur in the absence of maternal preeclampsia, particularly in late-onset disease.

The distribution observed here therefore appears broadly consistent with current understanding. The authors should clarify which specific paradigm or prior evidence is being challenged by their findings.

We thank the reviewer for this important comment and agree that fetal growth restriction (FGR) is currently recognized as a multifactorial condition, with many cases occurring in the absence of maternal preeclampsia, particularly in late-onset disease. Our intention was not to suggest that non-preeclamptic FGR is a novel concept, which has indeed been previously described in the literature and incorporated into current classifications of placental insufficiency and late-onset FGR.

We have therefore revised the Discussion to clarify that the paradigm being challenged is the persistent classical view that placental vascular malperfusion leading to FGR is predominantly linked to maternal preeclampsia or hypertensive disease. This concept is still reflected in many reviews and textbooks describing maternal vascular malperfusion as the principal mechanism underlying FGR in structurally normal fetuses.

Our findings do not contradict the multifactorial nature of FGR, but rather emphasize quantitatively that in a large Doppler-defined placental FGR population, the majority of cases (74%) occurred without clinically overt maternal preeclampsia, especially after 34 weeks’ gestation. We therefore believe that the key issue raised by our data is not the existence of non-preeclamptic FGR itself, but the relative predominance of maternal “tolerance” phenotypes (VFGR) over maternal systemic disease phenotypes (PFGR), despite apparently similar placental vascular insufficiency.

The manuscript has been revised accordingly to temper the wording and better distinguish between historical concepts of “maternal vascular malperfusion-associated FGR” and the contemporary understanding of heterogeneous placental FGR phenotypes.

WE HAVE ADDED A PARAGRAPH page 16.

Reviewer #2: In this cohort study attention was focused on placenta-related FGR, associated or not to Preeclampsia/eclampsia.

The large cohort suffers of time spanning. Neonatology markedly improved along years with outcome not simply comparable. Moreover, the definition of FGR are not granted to be obtained with same parameter and could allow heterogeneity during such a long period of data collection.

1. Being a single-center study, data collection took over 23 yrs period. Meanwhile, definition of placenta FGR have been changed. How the Authors accommodate their labelling according the to the last Delphi consensus.

We thank the reviewer for this important methodological comment.

First, regarding temporal changes in neonatal management, we acknowledge that obstetrical and neonatal practices evolved during the 24.5-year study period. However, the principal objective of the present study was not to compare neonatal survival across eras, but rather to compare maternal clinical characteristics and risk-factor profiles between preeclampsia-associated FGR (PFGR) and non-preeclamptic vascular FGR (VFGR). These maternal phenotypic associations are less likely to be substantially influenced by improvements in neonatal intensive care over time.

Second, we fully agree that definitions of placental fetal growth restriction (FGR) evolved during the long study period, particularly with the publication of the international Delphi consensus criteria for FGR diagnosis (our revised reference 14). However, our study was not designed as a retrospective reclassification of all pregnancies according to contemporary Delphi criteria. Instead, it reflects a real-world prospective clinical cohort in which cases labelled as “FGR” corresponded to pregnancies recognized and managed by obstetricians as placental vascular FGR during routine care.

Third, importantly, the study database started in January 2001, at a period when Doppler velocimetry had already become integrated into standard obstetrical evaluation of suspected placental insufficiency following the seminal studies of the late 1980s and 1990s. Throughout the study period, the diagnosis of FGR in our center relied on abnormal fetal growth trajectories associated with Doppler abnormalities and clinical surveillance for placental insufficiency, which is conceptually consistent with the pathophysiological framework later formalized by the Delphi consensus.

The Delphi procedure mainly standardized and refined diagnostic thresholds and combinations of biometric and Doppler criteria, particularly for early- versus late-onset FGR, rather than introducing an entirely new disease entity. Therefore, although exact Delphi variables were not prospectively coded in our database throughout the entire 24.5-year period, the pregnancies identified as VFGR or PFGR correspond clinically to what would currently be considered placenta-mediated Doppler-defined FGR. Importantly, FGR cases were not identified retrospectively from birthweight centiles alone, but from prospectively coded clinical indications recorded by physicians or midwives (labor induction, cesarean delivery, hospitalization in high-risk pregnancy units, prenatal diagnosis, etc.), thereby reflecting real-life clinical recognition of placental vascular FGR.

To address this issue transparently, we have added a paragraph in the “S

Attachment Submitted filename: RESPONSES TO REVIEWERS.docx

# Peer review: Decision Letter 1

**DOI:** 10.1371/journal.pone.0354687.r003

**Authors:** Umberto Simeoni

13 Jul 2026

Why Only One-Quarter of Placental Fetal Growth Restriction

Triggers Preeclampsia ? Clinical Risk Factors in 2060 Cases in a

24-Year Cohort of 90,000 births.

PONE-D-26-03357R1

Dear Dr. Robillard,

We’re pleased to inform you that your manuscript has been judged scientifically suitable for publication and will be formally accepted for publication once it meets all outstanding technical requirements.

Within one week, you’ll receive an e-mail detailing the required amendments. When these have been addressed, you’ll receive a formal acceptance letter and your manuscript will be scheduled for publication.

An invoice will be generated when your article is formally accepted. Please note, if your institution has a publishing partnership with PLOS and your article meets the relevant criteria, all or part of your publication costs will be covered. Please make sure your user information is up-to-date by logging into Editorial Manager at Editorial Manager® and clicking the ‘Update My Information' link at the top of the page. For questions related to billing, please contact billing support.

If your institution or institutions have a press office, please notify them about your upcoming paper to help maximize its impact. If they’ll be preparing press materials, please inform our press team as soon as possible -- no later than 48 hours after receiving the formal acceptance. Your manuscript will remain under strict press embargo until 2 pm Eastern Time on the date of publication. For more information, please contact onepress@plos.org.

Kind regards,

Umberto Simeoni

Academic Editor

PLOS One

Additional Editor Comments (optional):

Reviewers' comments:

Reviewer's Responses to Questions

**Comments to the Author**

Reviewer #1: All comments have been addressed

Reviewer #2: (No Response)

**********

2. Is the manuscript technically sound, and do the data support the conclusions?

Reviewer #1: Yes

Reviewer #2: Yes

**********

3. Has the statistical analysis been performed appropriately and rigorously?

Reviewer #1: Yes

Reviewer #2: Yes

**********

4. Have the authors made all data underlying the findings in their manuscript fully available?

The PLOS Data policy

Reviewer #1: No

Reviewer #2: Yes

**********

5. Is the manuscript presented in an intelligible fashion and written in standard English?

Reviewer #1: Yes

Reviewer #2: Yes

**********

Reviewer #1: I would like to thank the authors for their thorough and thoughtful responses to my comments. They have addressed all of my concerns clearly and with appropriate supporting references. The revisions have substantially improved the quality and clarity of the manuscript.

In particular, I found the additional discussion to be highly valuable. The authors provide well-reasoned and informative perspectives on the current debate surrounding the definition of fetal growth restriction, which considerably strengthens the manuscript and will be of interest to readers in the field.

Overall, I believe the manuscript has been significantly improved and I have no further comments.

Reviewer #2: every comment was adequately addressed and new revised paper was improved in clarity and consitency.

every every comment was adequately addressed and new revised paper was improved in clarity and consitency. was adequately every comment was adequately addressed and new revised paper was improved in clarity and consitency.every comment was adequately addressed and new revised paper was improved in clarity and consitency. and new revised paper was improved in clarity and consitency.

every comment was adequately addressed and new revised paper was improved in clarity and consitency.

**********

what does this mean?). If published, this will include your full peer review and any attached files.

If you choose “no”, your identity will remain anonymous but your review may still be made public.

**Do you want your identity to be public for this peer review?**  For information about this choice, including consent withdrawal, please see our Privacy Policy

Reviewer #1: **Yes:** Wen Lun Yuan

Reviewer #2: No

**********

# Editor report: Acceptance letter

**DOI:** 10.1371/journal.pone.0354687.r004

**Authors:** Umberto Simeoni

PONE-D-26-03357R1

PLOS One

Dear Dr. Robillard,

I'm pleased to inform you that your manuscript has been deemed suitable for publication in PLOS One. Congratulations! Your manuscript is now being handed over to our production team.

At this stage, our production department will prepare your paper for publication. This includes ensuring the following:

* All references, tables, and figures are properly cited

* All relevant supporting information is included in the manuscript submission,

* There are no issues that prevent the paper from being properly typeset

You will receive further instructions from the production team, including instructions on how to review your proof when it is ready. Please keep in mind that we are working through a large volume of accepted articles, so please give us a few days to review your paper and let you know the next and final steps.

Lastly, if your institution or institutions have a press office, please let them know about your upcoming paper now to help maximize its impact. If they'll be preparing press materials, please inform our press team within the next 48 hours. Your manuscript will remain under strict press embargo until 2 pm Eastern Time on the date of publication. For more information, please contact onepress@plos.org.

You will receive an invoice from PLOS for your publication fee after your manuscript has reached the completed accept phase. If you receive an email requesting payment before acceptance or for any other service, this may be a phishing scheme. Learn how to identify phishing emails and protect your accounts at https://explore.plos.org/phishing.

If we can help with anything else, please email us at customercare@plos.org.

Thank you for submitting your work to PLOS One and supporting open access.

Kind regards,

PLOS One Editorial Office Staff

on behalf of

Prof. Umberto Simeoni

Academic Editor

PLOS One
